# Supplementary material for: Machine learning enables detection of early-stage colorectal cancer by whole-genome sequencing of plasma cell-free DNA
Source: BMC Cancer. 2019 Aug 23;19:832. doi: 10.1186/s12885-019-6003-8 (PMC6708173; doi:10.1186/s12885-019-6003-8)
Supplement: Supplementary file 2 — Figure S1. Performance of confounding variable in k-fold and target CV procedure (ROC curves) for: a) Age and binned-age CV b) Batch and k-batch CV c) Processing date and ordered k-batch CV d) Institution and balanced k-batch CV. Note that stratification CVs (i.e., binned-age, k-batch, and ordered k-batch) perform at chance (mean AUC of 0.50) when the distribution of labels in the test covariate is even and would result in an ROC that is just a diagonal line. The variance seen in some of the target procedures is due to class imbalance in the test data. (DOCX 420 kb) [file 12885_2019_6003_MOESM2_ESM.docx]

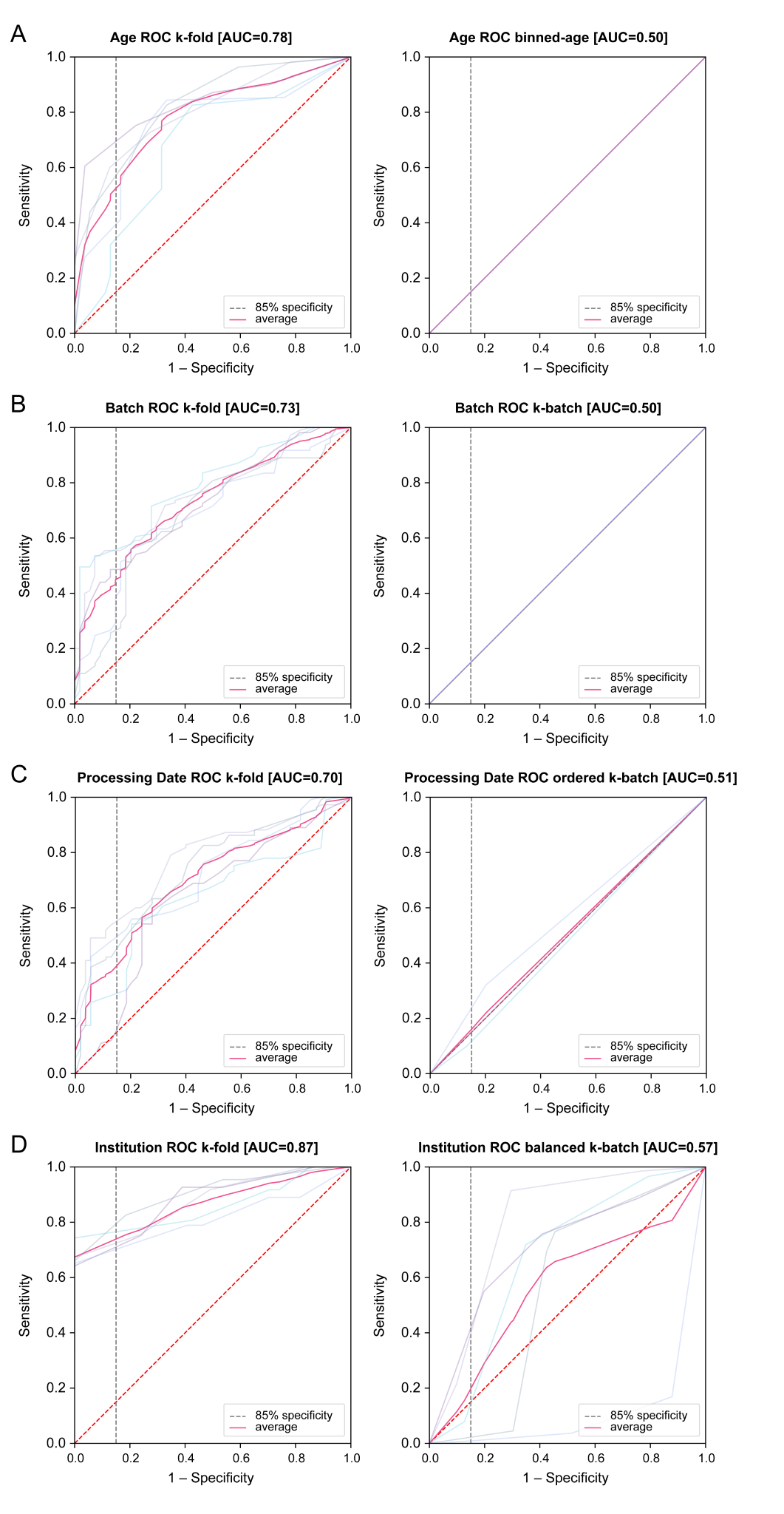


**Figure S1** Performance of confounding variable in k-fold and target CV procedure (ROC curves) for:

**a** Age and binned-age CV **b** Batch and k-batch CV **c** Processing date and ordered k-batch CV **d** Institution and balanced k-batch CV

Note that stratification CVs (i.e., binned-age, k-batch, and ordered k-batch) perform at chance (mean AUC of 0.50) when the distribution of labels in the test covariate is even and would result in an ROC that is just a diagonal line. The variance seen in some of the target procedures is due to class imbalance in the test data.
