# Supplementary material for: Machine learning enables detection of early-stage colorectal cancer by whole-genome sequencing of plasma cell-free DNA
Source: BMC Cancer. 2019 Aug 23;19:832. doi: 10.1186/s12885-019-6003-8 (PMC6708173; doi:10.1186/s12885-019-6003-8)
Supplement: Supplementary file 3 — Figure S2. Percentage of colorectal cancer samples by stage and non-cancer controls in each range of estimated TF (based on observed CNV using an IchorCNA-based estimate). (DOCX 74 kb) [file 12885_2019_6003_MOESM3_ESM.docx]

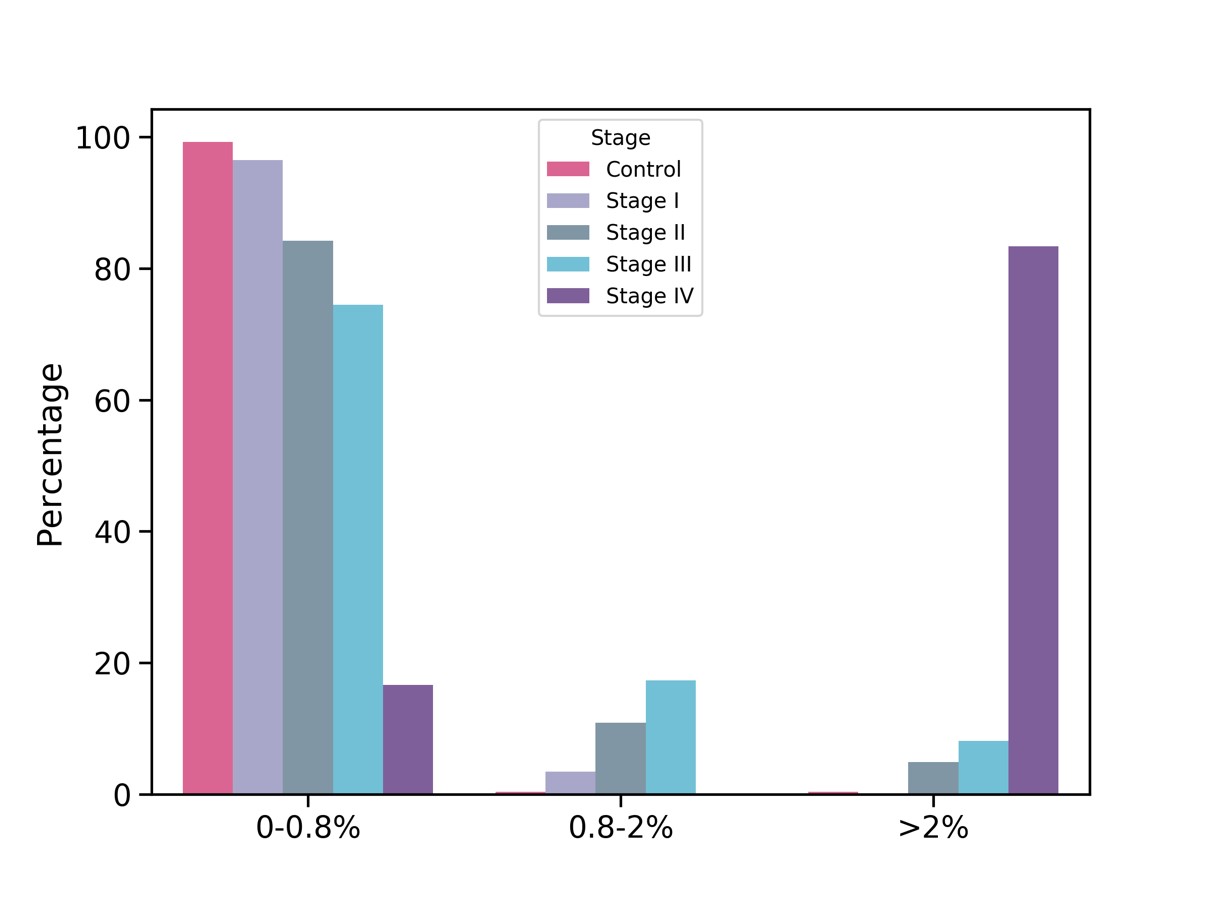


**Figure S2** Percentage of colorectal cancer samples by stage and non-cancer controls in each range of estimated TF (computed from observed CNVs using an IchorCNA-based estimate).
