# Supplementary material for: Machine learning enables detection of early-stage colorectal cancer by whole-genome sequencing of plasma cell-free DNA
Source: BMC Cancer. 2019 Aug 23;19:832. doi: 10.1186/s12885-019-6003-8 (PMC6708173; doi:10.1186/s12885-019-6003-8)
Supplement: Supplementary file 4 — Figure S3. IchorCNA-based estimated TF alone to predict case status (i.e., cancer or control) achieved an AUC of 0.67 in the IU age range, meaningfully lower than results from the ML model under any analyzed CV procedure. (DOCX 101 kb) [file 12885_2019_6003_MOESM4_ESM.docx]

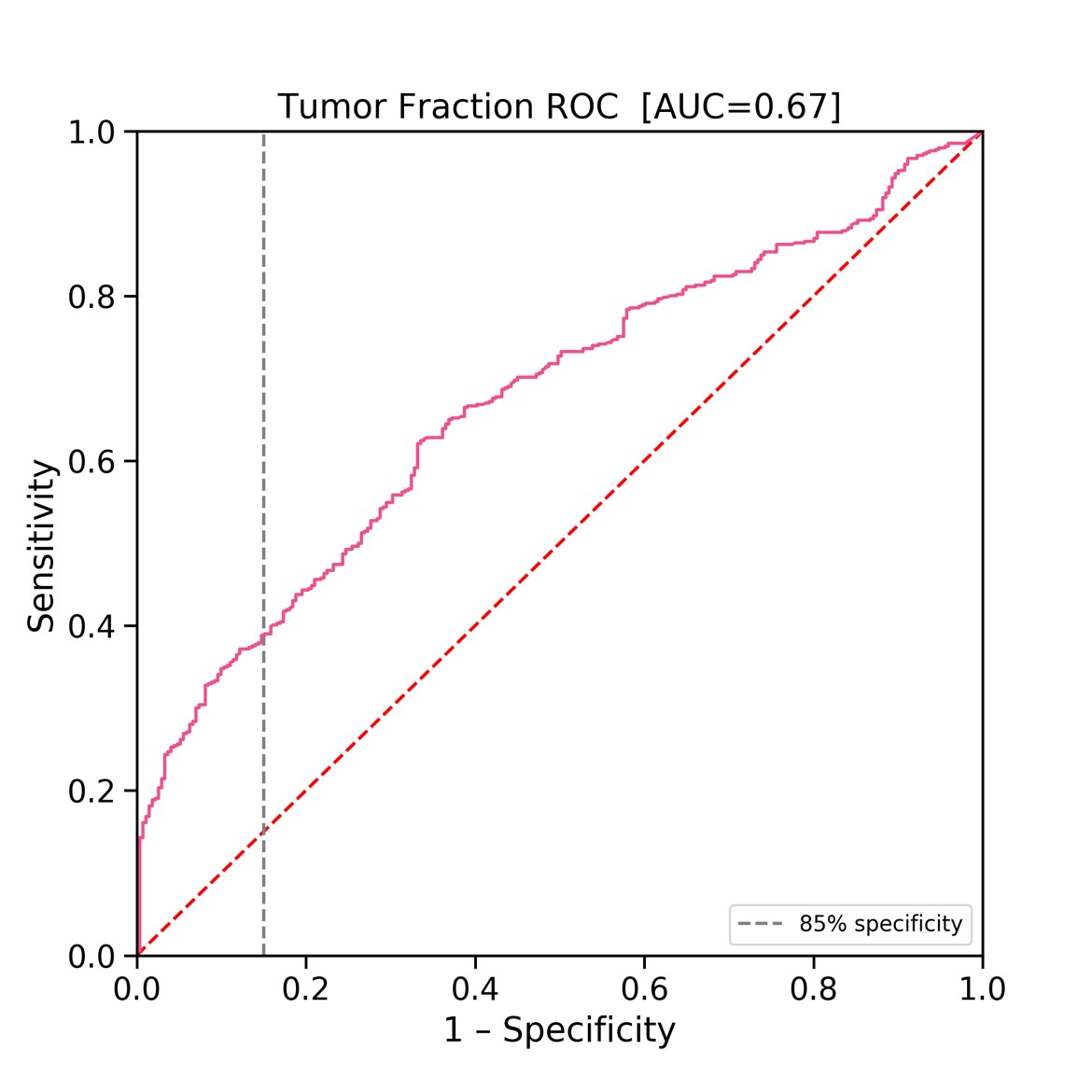


**Figure S3** IchorCNA-based estimated TF alone to predict cancer achieved an AUC of 0.67 in the IU age range, meaningfully lower than results from the ML model under any analyzed CV procedure.
