# Supplementary material for: Machine learning enables detection of early-stage colorectal cancer by whole-genome sequencing of plasma cell-free DNA
Source: BMC Cancer. 2019 Aug 23;19:832. doi: 10.1186/s12885-019-6003-8 (PMC6708173; doi:10.1186/s12885-019-6003-8)
Supplement: Supplementary file 5 — Figure S4. Non-linear relationship between the total number of samples used for training and sensitivity at 85% specificity for colorectal cancer detection. The method was trained again with k-fold, except the number of training samples per fold was downsampled. The lower numbers are comparable to those available for balanced k-batch and were used to investigate decreased classifier performance due to smaller sample sizes in training. (DOCX 60 kb) [file 12885_2019_6003_MOESM5_ESM.docx]

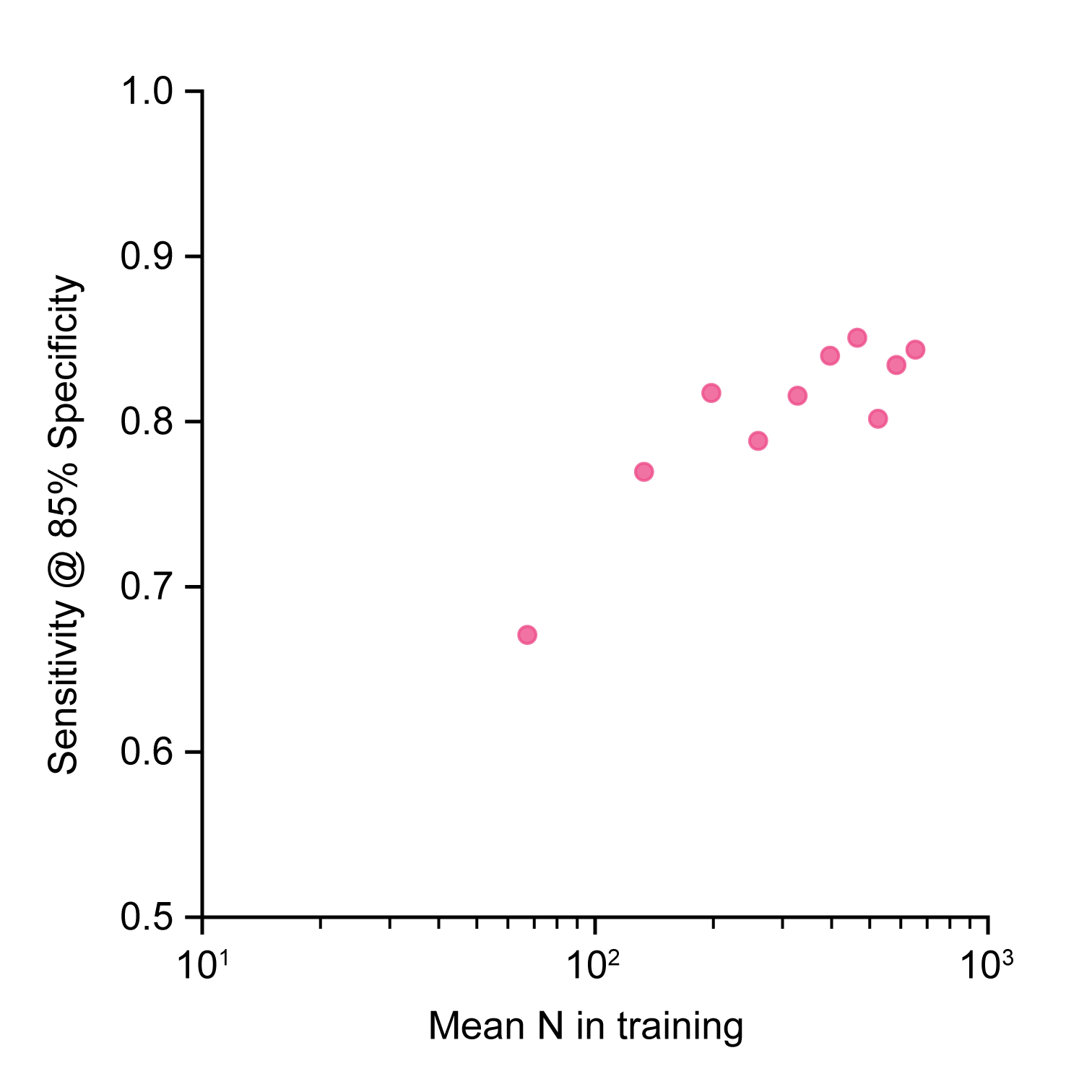


**Figure S4** Non-linear relationship between the total number of samples used for training and sensitivity at 85% specificity for colorectal cancer detection. The method selected by colorectal cancer analyses was trained again with k-fold except the number of training samples per fold were downsampled. The lower numbers are comparable to those available for balanced k-batch and were used to investigate decreased classifier performance due to smaller sample sizes in training.
