# Supplementary material for: Machine learning enables detection of early-stage colorectal cancer by whole-genome sequencing of plasma cell-free DNA
Source: BMC Cancer. 2019 Aug 23;19:832. doi: 10.1186/s12885-019-6003-8 (PMC6708173; doi:10.1186/s12885-019-6003-8)
Supplement: Supplementary file 6 — Table S1. Mean and standard deviation of the number of samples (both in and out of the IU age range) per fold for each CV procedure. SD = standard deviation. (DOCX 16 kb) [file 12885_2019_6003_MOESM6_ESM.docx]

**Table S1** Sample Sizes for Confounder CV Methods

| **Confounder CV Method** | **Number of Folds** | **Mean N in Training** | **SD of N in Training** | **Mean N in Test** | **SD of N in Test** |
| --- | --- | --- | --- | --- | --- |
| **k-fold** | 5 | 653.6 | 0.89 | 163.4 | 0.89 |
| **Binned-age** | 6 | 707.8 | 26.4 | 109.2 | 26.4 |
| **k-batch** | 5 | 653.6 | 35.0 | 163.4 | 35.0 |
| **Ordered k-batch** | 5 | 653.6 | 80.8 | 163.4 | 80.8 |
| **Balanced k-batch** | 5 | 263.6 | 39.0 | 163.4 | 35.0 |

Mean and standard deviation of the number of samples (both in and out of the IU age range) per fold for each CV procedure. SD = standard deviation
